# Dietary modulation of Lp(a): more questions than answers

**DOI:** 10.1016/j.jlr.2024.100592

**Published:** 2024-07-10

**Authors:** Penny M. Kris-Etherton, Terrence M. Riley, Kristina S. Petersen

**Affiliations:** 1 Department of Nutritional Sciences, Pennsylvania State University, University Park, PA, USA; 2 Nutritional Physiology Lab, Pennington Biomedical Research Center, Baton Rouge, LA, USA

Lipoprotein (a) [Lp(a)] is an independent causal risk factor for cardiovascular disease (CVD) that is primarily under genetic regulation. Lp(a) confers residual CVD risk and is considered a risk-enhancing factor (1). In the United States approximately one in five individuals have elevated Lp(a) (2). At present, expert opinion is that that diet has little to no effect on Lp(a) (3, 4). However, accumulating evidence suggests diet modestly modulates Lp(a) concentrations (5). Specifically, several clinical trials have shown saturated fat reduction increases Lp(a) concentrations. A recent meta-analysis of 27 randomized controlled trials including 1,325 participants demonstrated Lp(a) concentration was higher (standardized mean difference 0.14; 95% confidence interval 0.03, 0.24) after lower saturated fat diets than higher saturated fat diets (6). In the current issue of the *Journal of Lipid Research*, we see another example of dietary modulation of Lp(a) concentration.

In this issue, Law *et al.*, (7) report that consumption of 25% of energy requirements as glucose- or fructose-containing sugar-sweetened beverages (SSBs) that isocalorically replaced other dietary carbohydrate for 10 weeks reduced Lp(a) by ∼13% in adults with overweight/obesity, which was independent of apolipoprotein(a) [apo(a)] size variability. In addition, a 9% increase in low density lipoprotein cholesterol (LDL-C) was observed in this study with both sugar-containing beverages. This discordance in Lp(a) and LDL-C responses is also observed in studies of saturated fat reduction (5). The clinical implications and mechanisms remains unclear; however, it is known that Lp(a) synthesis and catabolism intersect in many ways with low density lipoprotein (LDL) metabolism. Shared pathways stem from the structural similarities between Lp(a) and LDL including an apolipoprotein B-100 (apoB-100), a lipid core, and a phospholipid membrane. Like apoB-100, apo(a) is synthesized in the liver. Apo(a) is combined with an apoB-100 intracellularly followed by covalent linkage after secretion (8). Upon secretion, Lp(a) and very low density lipoprotein (VLDL), a precursor of LDL, undergo delipidation and modification in peripheral circulation with overlapping receptor targets. The VLDL receptor has been shown to bind and internalize both particles (9) and scavenger receptor B1 contributes to cholesterol uptake. Removal of Lp(a) and LDL can occur by LDL receptors that internalize and degrade the lipid cores leading to lower circulating concentrations.

While Lp(a) and LDL metabolic pathways overlap, plasma concentrations sometimes show independent or opposite effects following dietary interventions, like observed by Law *et al.* (7) In the study by Law *et al.*, the increase in LDL-C in response to high sugar consumption is likely explained by an increase in fatty acid synthesis and VLDL secretion (10). This aligns with previously reported findings from this trial, which demonstrated hepatic de novo lipogenesis was increased following the fructose containing SSBs (11).

The reductions in Lp(a) observed with intake of the SSBs may relate to the metabolism of plasma apo(a). A clinical trial conducted in women with dyslipidemia showed a 2-fold greater residence time for plasma apo(a) than apoB-100 in the postprandial state suggesting apo(a) dissociation and recycling with apoB-100–containing particles (12). The ability of apo(a) to release and rebind apoB-100 lipoproteins may be inhibited by high circulating glucose concentrations and/or sugar intake due to greater LDL glycation potential that can impair lipoprotein lipase recognition and facilitate oxidative modification (13). Unrecycled plasma apo(a) that could result from high circulating glucose concentrations/high refined sugar intake would then be removed by other tissues thus lowering Lp(a) concentrations (see Fig. 1).

**Fig. 1:**
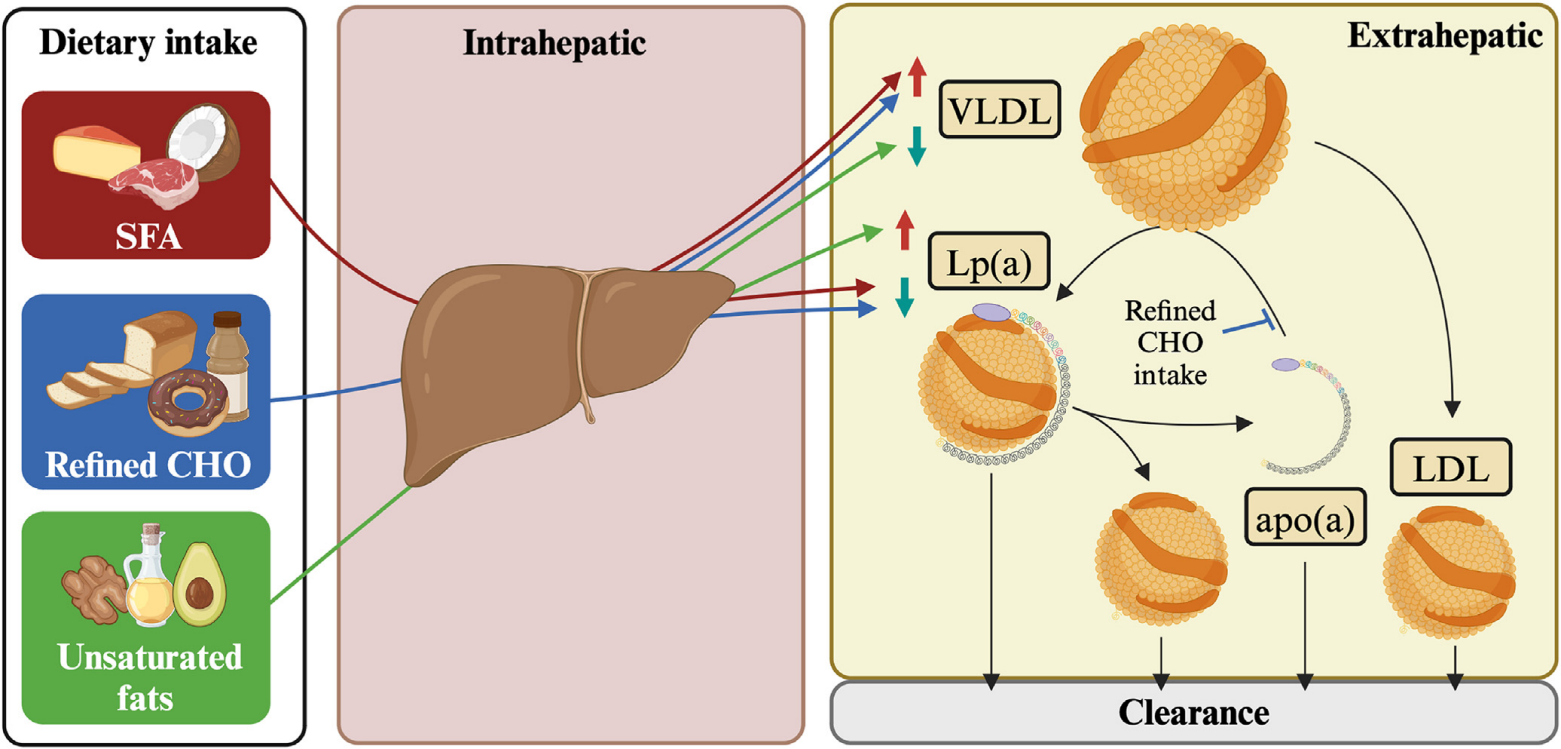
Divergent effects in LDL and Lp(a) concentrations following dietary interventions. Greater intake of SFA increases LDL but tends to decrease Lp(a). Replacing SFA with unsaturated fats can decrease LDL but increases Lp(a). Refined CHO can increase fatty acid synthesis and VLDL secretion and may decrease Lp(a) by inhibiting the Lp(a) recycling. Apo(a), apolipoprotein(a); CHO, carbohydrates; LDL, low density lipoprotein; PUFA, polyunsaturated fatty acids; SFA, saturated fatty acids; VLDL, very low density lipoprotein. Created with BioRender.com.

Interestingly, epidemiological and mendelian randomization studies show very low levels of Lp(a) are associated with increased risk of type 2 diabetes (14). Although causality remains uncertain, the study conducted by Law *et al.*, (7) may contribute to the understanding of this documented inverse relationship. In a previous publication of results from this trial, Stanhope *et al.*, (11) reported that the fructose containing SSBs increased total body weight, total body fat, visceral adiposity, fasting plasma glucose and insulin concentrations, and decreased insulin sensitivity after 10 weeks compared to baseline. The glucose containing SSBs increased total body weight, total body fat and subcutaneous adipose tissue, and increased glucose excursions following a 3-h oral glucose tolerance test after 10 weeks compared to baseline. These findings suggest type 2 diabetes risk was increased with both the glucose and fructose containing SSBs and coupled with the reductions observed in Lp(a) align with observational evidence linking very low Lp(a) concentrations to increased type 2 diabetes risk. Epidemiological evidence shows impaired fibrinolysis is associated with increased risk of type 2 diabetes (15, 16, 17, 18). It has been hypothesized that the plasminogen cascade has a role in the increased risk of type 2 diabetes observed with low Lp(a) concentrations (14). Further research is needed to clarify the role of low Lp(a) in the development of type 2 diabetes and examination of the plasminogen cascade in participants from this trial may be informative.

This study raises many questions about the role of diet in modulating Lp(a) and the relevance for CVD and type 2 diabetes risk. However, further research is needed to determine the clinical implications of Law *et al.* (7) findings for several reasons. First, current guidance is to measure Lp(a) once in a lifetime because it is considered genetically determined and stable. Secondly, the implications for disease risk of increasing or decreasing Lp(a) therapeutically or with lifestyle interventions is unknown. It is well documented that statins sometimes increase Lp(a) (2); however, statins are highly efficacious in reducing CVD risk (19). Finally, at baseline the cohort studied by Law *et al.*, (7) had an Lp(a) of 10.3 mg/dl, which is well below the cut point for low risk (<30 mg/dl) (2), and based on the current understanding of the association between Lp(a) and CVD risk would not be expected to meaningfully change CVD risk. Law *et al.*, (7) conducted an exploratory analysis examining if the Lp(a) response to the SSBs differed by baseline Lp(a) concentration and observed a similar magnitude of effect in participants with an Lp(a) concentration <30 mg/dl (n = 25; −12.9% ± 5.3) and ≥30 mg/dl (n = 7; −14.1% ± 6.0). In summary, research is needed to clarify the magnitude of dietary modulation of Lp(a) and implications for cardiometabolic disease risk.

## Conflict of interest

The authors declare that they have no conflicts of interest with the contents of this article.
